# Physiologic Responses to Infrarenal Aortic Cross-Clamping during Laparoscopic or Conventional Vascular Surgery in Experimental Animal Model: Comparative Study

**DOI:** 10.1155/2008/581948

**Published:** 2008-03-27

**Authors:** María F. Martín-Cancho, Verónica Crisóstomo, Federico Soria, Carmen Calles, Francisco M. Sánchez-Margallo, Idoia Díaz-Güemes, Jesús Usón-Gargallo

**Affiliations:** Minimally Invasive Surgery Centre Jesús Usón, Carretera Nac. 521, Km 41.8, 10071 Cáceres, Spain

## Abstract

The aim of this study was to compare the hemodynamic and ventilatory effects of prolonged infrarenal aortic cross-clamping in pigs undergoing either laparotomy or laparoscopy. 18 pigs were used for this study. Infrarenal aortic crossclamping was performed for 60 minutes in groups I (laparotomy, *n* = 6) and II (laparoscopy, *n* = 6). Group III (laparoscopy, *n* = 6) underwent a 120-minute long pneumoperitoneum in absence of aortic clamping (sham group). Ventilatory and hemodynamic parameters and renal function were serially determined in all groups. A significant decrease in pH and significant increase in PaCO_2_ were observed in group II, whereas no changes in these parameters were seen in group I and III. All variables returned to values similar to baseline in groups I and II 60 minutes after declamping. A significant increase in renal resistive index was evidenced during laparoscopy, with significantly higher values seen in Group II. Thus a synergic effect of pneumoperitoneum and aortic cross-clamping was seen in this study. These two factors together cause decreased renal perfusion and acidosis, thus negatively affecting the patient's general state during this type of surgery.

## 1. INTRODUCTION

Abdominal aortic surgery has improved greatly during the last 50 years, mainly thanks to advances in both anesthetic and surgical techniques. Abdominal laparoscopy is normally perceived to be associated with fewer risks [1]. However, clinicians should be aware of inherent dangers such as gaseous embolization, a potential inability to control hemorrhage, an increase in carbon dioxide arterial partial pressure, and changes in arterial blood pressure and heart rate. The hemodynamic and respiratory alterations associated with abdominal laparoscopy are caused by the high intra-abdominal pressure brought over by pneumoperitoneum creation. The most relevant hemodynamic changes are a decrease in venous return secondary to IVC compression and increases in central venous pressure and arterial blood pressure in absence of heart rate changes. Regarding respiratory adjustments, cranial displacement of the diaphragm causes a restrictive respiratory syndrome with decreased pulmonary compliance and increased pulmonary pressures and inspiratory peak [2, 3].

When used in the management of aortic diseases, the laparoscopic approach poses further complications and risks that must be addressed, such as the long aortic cross-clamping and surgical times [4]. Moreover, aortic cross-clamping in itself causes certain hemodynamic changes, whose effects must be taken into account along with the alterations secondary to laparoscopy. Previous human studies [4] reported severe problems during aortic declamping and very prolonged anesthetic recovery times.

Despite recent reports describing the feasibility of the laparoscopic approach for the management of aortic occlusive and aneurismatic disease [5–8] these studies focused on the surgical technique and its technical feasibility, without assessing hemodynamic and ventilatory stability or the patient's recovery from the procedure.

Data compiled in a review written by Gelman in 1995 [9] demonstrated that aortic cross-clamping and declamping are associated to severe disturbances in homeostasis in virtually all body systems. In experimental and clinical settings, cardiovascular responses to aortic cross-clamping are characterized by increases in proximal arterial pressure and systemic vascular resistance, while cardiac output decreases [9–12].

Prior studies by Byrne et al. [13] assessed physiological responses to laparoscopic aortobifemoral bypass surgery, while Alfonsi et al. [14] focused their works on the evaluation of cardiac function during intraperitoneal CO_2_ insufflation for aortic surgery. However, those studies did not evaluate the combination of the effects of aortic cross-clamping and pneumoperitoneum during aortic surgery. Moreover, the anesthetic protocol used in Byrne's study [13] is no longer in clinical use in hospitals nowadays.

We have found no studies evaluating pathophysiologic changes secondary to cross-clamping and declamping of the abdominal aorta during vascular laparoscopic surgery. With this in mind, we consider that the evaluation of physiologic changes occurring during infrarenal aortic surgery, whether using conventional surgery or laparoscopic techniques, is extremely important in order to determine whether the observed hemodynamic changes can be attributed to pneumoperitoneum or aortic cross-clamping alone or to the combined effect of these two factors. Understanding these alterations will enable clinicians to improve the quality of anesthesia when performing this kind of surgery. In our opinion, laparoscopic aortic cross-clamping may cause physiological changes precluding the safe use of this technique in all patients.

## 2. MATERIALS AND METHODS

### 2.1. Animals

The experimental protocol was approved by the Institutional Ethical Committee for Animal Research. Eighteen healthy female large white pigs were used. Mean weight was 30.8 ± 2.0 kg.

### 2.2. Anesthesia

One day prior to the experimental anesthetic episode, each pig was anesthetized with sevoflurane (Sevorane, Abbott Laboratories, Madrid, Spain), and a 20-standard wire gauge catheter was placed in the carotid artery and fixed to the skin. Similarly, on that same day a 4Fr. vascular sheath was placed in the external jugular vein using the Seldinger technique. Pigs were randomly assigned to 3 groups. Groups I (*n* = 6) and II (*n* = 6) underwent aortic cross-clamping through either laparotomy in Group I or laparoscopy in Group II. Animals belonging to Group III underwent a 120-minute long laparoscopy in absence of aortic intervention (sham group). Food, but not water, was withheld for 8 hours prior to surgery. Body temperature was maintained stable at 37-38^*º*^C, using a thermal blanket (Astopad system, Stihler electronic GMBH. Stuttgart, Germany) and administering tempered fluids.

Animals were premedicated with IM diazepam (Valium, Roche Farma, Madrid, Spain) (0.1 mg/kg) and ketamine (Ketolar, Pfizer, Madrid, Spain) (10 mg/kg). Anesthesia was induced with propofol (4 mg/kg), which was administered after oxygenation by facial mask with 100% oxygen for 3 minutes. When lack of jaw tone, loss of swallowing, lack of head shaking, loss of palpebral and pain reflexes, and ventromedial rotation of the eyes were all detected, endotracheal intubation was performed with the tube connected to a semiclosed circular anesthetic circuit attached to a ventilator (Ventilator 7800, Ohmeda, Madrid, Spain). Sevoflurane administration started at 5%, which enabled us to rapidly achieve 1.25 MAC (1MAC = 2.66%) [10] with an oxygen flow rate of 3 L/min. Once 1.25 MAC was reached (3.3% Et sevoflurane), the vaporizer setting was adjusted as needed to maintain this concentration. Muscle relaxation was obtained by injection of vecuronium (0.1 mg/kg) (Norcuron, Organon Española, Barcelona Spain) every 30 minutes. A bolus (5 *μ*g/kg) of fentanyl (Fentanest, Roche, Madrid, Spain) was administered every 30 minutes. Systemic heparinization (Heparina Rovi 5%, laboratorios farmacéuticos Rovi SA, Madrid, Spain) was administered (150 UI/kg) 5 minutes prior to aortic cross-clamping. Postoperative analgesia was assured by administering 10 *μ*g/Kg of IM Buprenorphine (Buprex, Schering-Plough, Madrid, Spain) every 8 hours.

Intermittent positive pressure ventilation was used during the procedure to maintain end-tidal CO_2_ concentration between 35 to 45 mmHg, with a tidal volume of 10 mL/kg in the open surgery group and ranging between 13–15 mL/kg in the laparoscopy groups.

During surgery, continuous infusion of Ringer-lactate solution (Ringer-lactato, Braun, Barcelona, Spain) at a rate of 10 mL/kg/h was administered. 500 mL of a colloid solution (hydroxyethyl starch, Voluven 6%, Fresenius Kabi, Barcelona, Spain) was administered five minutes before declamping in every animal. At the end of the surgery, the vaporizer was switched off and fresh gas flow rate was increased to 10 L/min of 100% oxygen. Pigs were extubated when they regained swallowing reflexes and considered recovered and fully conscious when the attending anesthesiologists recorded their ability to stand and walk.

### 2.3. Surgical procedures

For pigs undergoing laparotomy, a standard ventral midline approach was used (Group I). For pigs undergoing laparoscopy (Groups II and III), 3 10 mm laparoscopic ports were inserted. Pneumoperitoneum was created by insufflating CO_2_ into the abdominal cavity; intra-abdominal pressure was maintained at 12 to 14 mm Hg in both groups (Groups II and III). In Group III, CO_2_ pneumoperitoneum was maintained for 120 minutes, which was considered the minimum time needed to complete the cross-clamping procedure performed in Group II.

In animals belonging to Groups I and II, aortic dissection was performed from the origin of the renal arteries to the origin of the caudal mesenteric artery, and all lumbar arteries in the target aortic segment were temporarily occluded. The aorta was then cross-clamped immediately below the origin of the caudal renal artery and immediately cranial to the inferior mesenteric artery. Aortic occlusion was maintained for 60 minutes.

These procedures were always performed by the same surgical team using the same technique in order to achieve reproducible stimulation.

### 2.4. Monitoring

BIS (A-1050TM, version 3.05.05, Aspect Medical Systems Inc, Natick, Mass, USA) was registered using a previously described electrodes montage [15]. Electrocardiography (Hewlett Packard model 86S, Hewlett Packard, Geneva, Switzerland) (lead II) and pulse oximetry with a probe (Clip Tip sensor, Oximeter Sensor, Datex-Ohmeda, Louisville, Colo, USA) placed on the tongue were monitored. Other parameters registered were rectal temperature, tidal volume, end-tidal concentration of sevoflurane, end-tidal CO_2_ concentration, and respiratory rate (Ohmeda RGM 5250, Ohmeda, Madrid, Spain). Muscle relaxation was observed and monitored by train-of-four (TOF) (TOF-Guard, Biometer International A/S, Odense, Denmark).

Arterial blood pressure, central venous pressure and heart rate were also measured using a blood pressure module (Hewlett Packard Press M 1006B, Hewlett Packard, Geneva, Switzerland) connected to a system for monitoring hemodynamic variables.

We measured hemodynamic variables in real time using the PulseCO continuous cardiac output monitoring system (LiDCO Ltd): cardiac output (CO), stroke volume (SV) and systemic vascular resistance (SVR). The system was calibrated using the lithium dilution technique [16] for cardiac output measurement at a lithium dosage of 0.04 mL/kg.

### 2.5. Arterial blood gasometry assays

Through the carotid artery, arterial blood was sampled at different times (see below). At each interval, 0.5 mL of blood were collected using a prefilled heparin syringe. pH, PaCO_2_, PaO_2_ and bicarbonate (CO3H-) were measured using the arterial gas analyzer (Radiometer Medical, model ABL77, Copenhagen, Denmark).

### 2.6. Renal function tests

Urine production was registered every 30 minutes, along with seric urea and creatinine. The Pourcelot or renal resistive index was also determined at these times (RI = peak systolic velocity − end diastolic velocity/peak systolic velocity) at the arcuate arteries of the corticomedullary junction, using a Panther 2002 (B&K Medical, Herlev, Denmark) ultrasound scanner with a conventional probe (5,5 MHz) for transcutaneous examination and a 9.8 mm laparoscopic probe (6,5 MHz) for the laparoscopic approach.

### 2.7. Data processing

All data were expressed as mean ± SD at the following times:

T1 baseline (immediately after connecting the patient to the monitoring systems prior to surgery;
T2 Groups I and II, 5 minutes before cross-clamping. Group III pneumoperitoneum establishment;
T3 Groups I and II, 30 minutes after cross-clamping. Group III 60 minutes after pneumoperitoneum creation;
T4 Groups I and II, 60 minutes after cross-clamping. Group III, 120 minutes after pneumoperitoneum creation;
T5 Groups I and II, 5 minutes after declamping or 5 minutes after the end of pneumoperitoneum in Group III;
T6 Groups I and II, 30 minutes after declamping or 30 minutes after the end of pneumoperitoneum in Group III;
T7 Groups I and II, 60 minutes after declamping or 60 minutes after the end of pneumoperitoneum in Group III.

A Kolmogorov Smirnov test [17] was used to determine that data were normally distributed. Changes in ventilatory and hemodynamic variables at each time point were analyzed using ANOVA for repeated measures followed by the Tukey test to examine intergroup deviation from control values. Recovery times were analyzed by use of ANOVA, using the group (laparotomy with cross-clamping, laparoscopy with cross-clamping, or laparoscopy alone) as the independent variable. Values of *P* < .05 were considered significant (SPSS 14.0 statistical package for Windows, SPSS Inc, Chicago, Ill, USA).

## 3. RESULTS

Mean ± SD duration of anesthesia was 216 ± 15 minutes in the animals that underwent laparotomy, 367 ± 24 minutes in the laparoscopic cross-clamping group and 195 ± 26 minutes in Group III. The difference between Groups I and II was mainly attributable to the different time needed to complete aortic dissection (12 ± 4 minutes for pigs undergoing laparotomy versus 39 ± 4 minutes for pigs undergoing laparoscopy) plus the time needed for placing the lumbar clips and the aortic clamps.

SpO_2_ was >97% and EtCO_2_ was maintained between 35 to 40 mmHg in all pigs. Blood loss was minimal for all procedures.

Anesthetic depth, as determined by BIS [18] and clinical observation was consistent with a surgical plane of anesthesia.

The changes observed in all the measured variables are reflected in Tables 1, 2, 3, 4, 5, and 6. In brief, these changes are summarized below.

Immediate and significant increases in ABP and SRV and decreases in CO and SV following aortic cross-clamping, without any concurrent significant changes to CVP or heart rate, were seen in Groups I and II (Tables 1, 2, and 3). After declamping (T5), a significant increase in CO and SV and a significant decrease in ABP and SVR were also observed.

In Groups II and III, pneumoperitoneum caused a significant increase in CVP, ABP, and SVR along with decreased CO (Tables 1, 2, and 3). In Group II a significant increase in PaCO_2_ and a significant decrease in pH (Table 4) were seen.

No significant changes in pH or arterial gasometry were caused by clamping or declamping in Group I, whereas in Group II a further significant decrease in pH values was seen during cross-clamping, whilst HCO3 was kept over 26 mmol/l during cross-clamping and declamping in order to compensate for the acidosis (Tables 4 and 5).

No significant changes in urea or creatinine were evidenced between groups. However, significant (*P* < .05) increases in the RI were seen during infrarenal aortic cross-clamping in Group II (laparoscopy) (Table 6) and during pneumoperitoneum creation in Group III, with the increase being significantly greater in Group II than in Group III. Similarly, urine production was decreased by 35% during laparoscopic clamping when compared to cross-clamping by open surgery, and by 30% during laparoscopic cross-clamping when compared to laparoscopy in absence of aortic cross-clamping.

60 minutes after declamping, all the studied variables had returned to baseline values in all groups. No significant differences were observed between groups in regard to recovery times except for the time to standing which was significantly lower in Groups II and III (pigs that had undergone laparoscopy) (Table 7).

## 4. DISCUSSION

While the systemic cardiovascular consequences of infrarenal aortic cross-clamping during aortic abdominal surgery are well documented in both humans [9] and pig [19], and have been reported to be very similar (this animal model reproduces the changes observed in humans), its repercussions during laparoscopic surgery have not been reported.

In the present study, pneumoperitoneum caused a significant increase in arterial blood pressure and systemic vascular resistance. Generally speaking, the higher arterial blood pressure observed may be attributed to the increase in intra-abdominal pressure caused by pneumoperitoneum, which gives rise to increased systemic vascular resistance and arterial blood pressure to compensate for the decrease in cardiac output secondary to the decreased venous return [2]. However other authors have reported that neither increased intra-abdominal pressure nor plasma accumulation of carbon dioxide influences cardiac output [20, 21]. In this study, a decrease in cardiac output was evidenced immediately after pneumoperitoneum creation. In our opinion, hypertension may also be caused by any of three factors: mechanical compression of the splanchnic vascular bed; a sympathetic reflex from the splanchnic regions; and the release of humoral vasoconstriction mediators, such as renin or vasopressin [21–23]. Systemic arterial hypertension has consistently been found during inflation of the peritoneum with CO_2_, but Huang et al. [24] reported that hypercarbia might not be the major determinant factor of it.

Cross-clamping of the aorta also caused a marked increase in arterial blood pressure, most likely due to the sudden increase in impedance to aortic blood flow and the resultant increase in systolic ventricular wall tension or afterload. However, factors such as myocardial contractility, preload, blood volume, and sympathetic nervous system activation may also be important [25].

Although several clinical reports have noted no significant hemodynamic response to infrarenal cross-clamping [26, 27], the hemodynamic response generally consists of increases in arterial pressure (7 to 10%) and systemic vascular resistance (20 to 32%) with no significant change in heart rate [9–11, 28]. Cardiac output is generally decreased by 9% to 33% [27]. Reported changes in ventricular filling pressures have been inconsistent [12, 27, 29]. Changes in these parameters seen in both groups, in the present study, followed a similar pattern.

Despite the increase in ABP and SVR and the decrease in CO brought over by pneumoperitoneum creation, these variables' values were similar in Groups I and II during aortic cross-clamping. In our opinion, aortic cross-clamping did not greatly affect hemodynamic changes secondary to pneumoperitoneum, as shown by the fact that these variables changed in absence of cross-clamping in Group III.

Regarding ventilatory changes, it is known that if correct ventilatory support is provided, SpO_2_ can easily be maintained >90% during laparoscopy. Carbon dioxide pneumoperitoneum causes absorption of this gas, and if lung ventilation is insufficient to eliminate the absorbed carbon dioxide, hypercapnia will develop, which may cause acidosis, depress myocardial function, and induce arrhythmias and cardiovascular collapse [2]. In view of this possibility, controlled ventilation was used in the present study to prevent hypercapnia, and minute ventilation was adjusted to maintain EtCO_2_ at 35–40 mmHg throughout the entire procedure, increasing by approximately 30% (minute ventilation before laparoscopy was 3.6 ± 0.2 L/min, and it reached 4.9 ± 0,5 L/min during laparoscopy) and maintaining a ventilatory rate of 10–12 breaths/min, as has been previously reported [30, 31]. Prior studies [20] reported that insufflation of the peritoneal cavity with CO_2_ to an intra-abdominal pressure <15 mmHg does not interfere significantly with pulmonary gas exchange in patients without preexisting cardiopulmonary diseases. Other authors reported a statistically significant correlation between PaCO_2_ and EtCO_2_ during 60 minutes of CO_2_ insufflation [32]. A significant decrease in pH and concomitant increase in PaCO_2_ were seen in Group II after clamping, whereas they remained stable in the other two groups at the same time points. These parameters remained changed in this group whilst the aorta was clamped, and they did not regain baseline values until 30 minutes (pH) or 60 minutes (PaCO_2_) after declamping. This could be attributed to the combined effect of aortic clamping and pneumoperitoneum, which could lead to the development of an acidosis of mixed metabolic and respiratory origin. The acidosis seen in Group II was not compensated by the above described ventilatory changes, therefore causing increased bicarbonate values and leading us to consider this mixed metabolic and hypercarbic origin for the acidosis, supporting the prior studies of Tobias et al. [33].

An increase in the partial pressure of arterial carbon dioxide was evidenced in this study. In our opinion, it can be due to CO_2_ absorption, rather than to the mechanical ventilatory repercussions of increased intra-abdominal pressure [34–36]. It has been previously described that, in healthy patients, absorption of CO_2_ from the abdominal cavity represents the main (or the only) mechanism responsible for increased PaCO_2_ [37]. However, in cases of cardiorespiratory compromise, ventilatory changes also contribute significantly to increasing PaCO_2_ [38]. Therefore, although increased PaCO_2_ may be well tolerated by young and healthy patients, the extent to which hypercapnia is acceptable has not been determined and probably varies according to the patient's physical status. It is thus wise to maintain PaCO_2_ within physiologic ranges by adjusting controlled mechanical ventilation [39].

Acute renal failure requiring dialysis is a severe complication in up to 5% of open aortic surgeries, and it causes high mortality rate [40]. No data about renal failure during laparoscopic aortic surgery could be found in the literature. However, the increased RI evidenced during laparoscopy in this study, both with and without aortic clamping, suggests that its incidence may be higher in this approach than in conventional aortic surgery. There are multiple factors involved in renal failure. On the one hand, aortic cross-clamping elicits marked hemodynamic changes that impact kidney function, increasing renal vascular resistance and markedly decreasing blood flow to the renal cortex [37]. On the other hand, the increased intra-abdominal pressure secondary to CO_2_ insufflation markedly contributes to the significant decrease in renal flow [41], due to vascular compression at the renal parenchyma and the decreased cardiac output seen in the groups undergoing laparoscopy. Despite the absence of permanent renal injury after aortic cross-clamping in either group, careful patient selection for procedures involving laparoscopic aortic cross-clamping is warranted, specially in patients presenting with preexisting nephropathies to avoid further deterioration of their condition.

Recovery was swift in the present study. Mean time to standing was significantly lower in Groups II and III (laparoscopy). This is probably attributable to a lesser degree of postoperative pain in animals subjected to laparoscopic surgery, as suggested by the fact that patients subjected to a laparotomy generally complain more of parietal pain (abdominal wall) whereas after laparoscopy, visceral pain is normally present [42].

To our knowledge, we report the first study concerning the physiologic changes occurring during infrarenal aortic surgery using laparoscopic technique. Hemodynamic management of aortic cross-clamping was similar for both surgical approaches in pig [43], however, aortic cross-clamping during laparoscopy causes mixed respiratory and metabolic acidosis that needs to be monitored by arterial gas analysis, even in the absence of abnormal EtCO_2_ levels, along with an increasing renal vascular resistance and markedly decreasing blood flow to the renal cortex. Despite being compensated and well tolerated in healthy pigs, this acidosis must be corrected by adjusting controlled mechanical ventilation. Further studies in experimental animal models [44] are required to determine the clinical implication of these findings and their impact in the presence of cardiovascular comorbidity (aortic diseases, hypertension, coronary artery disease, chronic obstructive pulmonary disease, etc.) during aortic surgery.

## Figures and Tables

**Table 1:** Heart rate and mean arterial blood pressure in pigs anesthetized with sevoflurane, fentanyl, and vecuronium and undergoing aortic cross-clamping through laparotomy (Group I) or laparoscopy with (Group II) or without aortic cross-clamping (Group III).

	Heart rate (beats/min)			Mean arterial blood pressure (mmHg)		
Times	Group I	Group II	Group III	Group I	Group II	Group III
T1	79.6 ± 11.7	81.6 ± 11.0	76.5 ± 3.1	68.2 ± 7.3	57.6 ± 1.8	65.7 ± 10.6
T2	78.8 ± 14.4	85.8 ± 12.3	78.5 ± 4.2	65.0 ± 7.4	73.0 ± 6.4*	81.7 ± 10.1*
T3	78.7 ± 14.4	86.8 ± 14.8	78.7 ± 4.1	78.8 ± 13.8*	77.0 ± 9.1*	84.3 ± 8.5*
T4	82.8 ± 18.7	88.0 ± 18.0	78.0 ± 6.2	83.2 ± 15.8*	78.8 ± 8.2*	85.2 ± 7.5*
T5	89.8 ± 21.2	100.0 ± 22.7	78.5 ± 7.0	52.6 ± 12.0*	61.8 ± 9.3	76.0 ± 15.7
T6	90.7 ± 15.3	94.0 ± 16.2	80.2 ± 8.3	64.7 ± 7.7	52.6 ± 4.9	68.8 ± 18.1
T7	93.0 ± 15.3	91.4 ± 11.9	78.3 ± 7.4	64.8 ± 8.0	53.6 ± 5.4	68.7 ± 16.2

Data are expressed as mean ± SD.

*Significant changes from baseline (*P* < .05).

**Table 2:** Central venous pressure (CVP), cardiac output (CO) in pigs anesthetized with sevoflurane, fentanyl, and vecuronium and undergoing aortic cross-clamping through laparotomy (Group I) or laparoscopy with (Group II) or without aortic cross-clamping (Group III).

	CVP (cm H_2_O)			CO (L/min)		
Times	Group I	Group II	Group III	Group I	Group II	Group III
T1	5.3 ± 1.4	3.6 ± 0.5	4.0 ± 0.5	4.16 ± 0.61	4.06 ± 0.62	3.57 ± 0.48
T2	5.0 ± 1.8	6.4 ± 3.2*	7.3 ± 1.2*	4.13 ± 0.52	3.49 ± 0.73	3.08 ± 0.36
T3	4.2 ± 2.1	6.2 ± 3.4*	6.6 ± 0.4*	4.12 ± 1.04	2.80 ± 1.21*	2.67 ± 0.15*
T4	5.8 ± 2.7	4.8 ± 2.5	5.5 ± 0.6	3.40 ± 1.21	3.12 ± 1.42*	2.88 ± 0.26
T5	4.8 ± 3.2	4.8 ± 3.5	3.4 ± 1.6	6.50 ± 2.12*	5.50 ± 1.88*	3.18 ± 0.40**^†^**
T6	5.5 ± 3.0	1.6 ± 0.5	2.7 ± 0.9	5.28 ± 1.67	4.88 ± 1.86	3.40 ± 0.67**^†^**
T7	5.8 ± 2.6	2.6 ± 1.5	2.6 ± 1.5	4.90 ± 1.11	4.54 ± 1.01	3.45 ± 0.60**^†^**

Data are expressed as mean ± SD.

*Significant changes from baseline (*P* < .05).

**Table 3:** Systolic volume (SV) and and systemic vascular resistance (SVR) in pigs anesthetized with sevoflurane, fentanyl, and vecuronium and undergoing aortic cross-clamping through laparotomy (Group I) or laparoscopy with (Group II) or without aortic cross-clamping (Group III).

	SV (mL)			SVR (Dyn s/cm^5^)		
Times	Group I	Group II	Group III	Group I	Group II	Group III
T1	49.0 ± 10.5	42.2 ± 20.0	42.5 ± 0.5	1556 ± 370	1460 ± 359	1735 ± 202
T2	45.8 ± 9.7	35.2 ± 10.3	41.5 ± 3.5	1509 ± 394	1940 ± 529*	2098 ± 133*
T3	38.0 ± 11.1	37.2 ± 13.6	36.8 ± 2.6	2083 ± 456*	2080 ± 957*	2455 ± 397*
T4	39.5 ± 15.4	38.0 ± 12.6	36.0 ± 2.4	2100 ± 610*	2010 ± 1016*	2508 ± 469*
T5	62.2 ± 25.4*	62.0 ± 33.3*	44.0 ± 5.5	933 ± 513*	1160 ± 290	1926 ± 410
T6	51.5 ± 17.7	43.0 ± 33.9	41.0 ± 1.5	1208 ± 483	1230 ± 256	2032 ± 482
T7	52.3 ± 14.5	45.6 ± 15.9	42.7 ± 1.9	1361 ± 636	1320 ± 97	1880 ± 337

*Significant changes from baseline (*P* < .05).

**Table 4:** Acid-base balance in pigs anesthetized with sevoflurane, fentanyl, and vecuronium and undergoing aortic cross-clamping through laparotomy (Group I) or laparoscopy with (Group II) or without aortic cross-clamping (Group III).

	pH			CO3H- (mmol/L)		
Times	Group I	Group II	Group III	Group I	Group II	Group III
T1	7.48 ± 0.01	7.47 ± 0.01	7.40 ± 0.73	26.7 ± 3.3	31.2 ± 1.1	30.1 ± 0.9
T2	7.43 ± 0.02	7.32 ± 0.07	7.40 ± 0.04	23.1 ± 7.3	27.4 ± 5.7^×^	30.0 ± 1.4^×^
T3	7.46 ± 0.04	7.27 ± 0.16^∗†^	7.40 ± 0.5	20.6 ± 4.3	31.2 ± 3.5^×^	31.0 ± 1.5^×^
T4	7.46 ± 0.05	7.28 ± 0.17^∗†^	7.40 ± 0.1	26.6 ± 2.3	33.8 ± 2.3^×^	30.8 ± 1.3^×^
T5	7.40 ± 0.06	7.22 ± 0.18^∗†^	7.43 ± 0.1	24.1 ± 3.4	29.1 ± 7.1^×^	30.3 ± 1.7^×^
T6	7.41 ± 0.06	7.34 ± 0.07	7.38 ± 0.6	24.7 ± 3.2	32.8 ± 3.6^×^	30.0 ± 1.6^×^
T7	7.38 ± 0.01	7.32 ± 0.07	7.34 ± 0.1	21.5 ± 7.6	32.5 ± 4.0^×^	30.3 ± 1.6^×^

*Significant changes from baseline (*P* < .05).

^†^Significantly (*P* < .05) different from values for pigs undergoing laparoscopy and cross-clamping.

^×^Significantly (*P* < .05) different from values for pigs undergoing laparoscopy.

**Table 5:** Acid-base balance in pigs anesthetized with sevoflurane, fentanyl, and vecuronium and undergoing aortic cross-clamping through laparotomy (Group I) or laparoscopy with (Group II) or without aortic cross-clamping (Group III).

	PaCO_2_ (mmHg)			PaO_2_ (mmHg)		
Times	Group I	Group II^†^	Group III	Group I	Group II	Group III
T1	36.0 ± 5.0	41.1 ± 3.7	38.0 ± 2.4	487.8 ± 99.3	574.2 ± 81.1	501.2 ± 41.7
T2	33.0 ± 9.2	45.6 ± 6.4	43.0 ± 6.5	447.3 ± 111.7	370.6 ± 88.2*	495.7 ± 37.3
T3	30.7 ± 6.1	48.1 ± 3.8*	43.0 ± 3.9	443.7 ± 34.8	399.8 ± 96.3*	479.0 ± 33.2
T4	37.5 ± 6.9	51.6 ± 2.3*	43.7 ± 3.0	501.7 ± 64.9	392.6 ± 110.5*	456.7 ± 48.6
T5	39.5 ± 7.2	49.0 ± 4.4*	42.2 ± 2.2	497.0 ± 58.2	458.2 ± 106.2	445.0 ± 47.6
T6	37.8 ± 7.0	48.6 ± 4.0*	39.2 ± 2.2	481.0 ± 72.7	440.0 ± 105.4	448.7 ± 26.6
T7	38.5 ± 8.5	46.0 ± 2.2	38.0 ± 2.3	434.3 ± 76.9	452.0 ± 106.9	462.0 ± 16.9

Data are expressed as mean ± SD.

^†^Significantly (*P* < .05) different from values obtained for pigs undergoing laparoscopy and cross-clamping.

*Significant changes from baseline within each group (*P* < .05).

**Table 6:** Renal resistive index values obtained thorough the study.

	T1	T2	T3	T4	T5	T7
Group I	0.48 ± 0.03	—	0.58 ± 0.11	0.63 ± 0.07	0.56 ± 0.10	0.53 ± 0.10
Group II	0.53 ± 0.10	0.69 ± 0.05^∗†^	0.69 ± 0.06^∗†^	0.68 ± 0.08^∗†^	0.68 ± 0.02^∗†^	0.56 ± 0.02
Group III	0.49 ± 0.03	0.58 ± 0.03*	0.58 ± 0.11*	0.64 ± 0.03*	0.60 ± 0.10*	0.49 ± 0.02

^†^Significantly (*P* < .05) different from values obtained for pigs undergoing laparoscopy and cross-clamping.

*Significant changes from baseline within each group (*P* < .05).

**Table 7:** Recovery times in pigs anesthetized with sevoflurane, fentanyl, and vecuronium and undergoing aortic cross-clamping through laparotomy (Group I) or laparoscopy with (Group II) or without aortic cross-clamping (Group III).

	Recovery time (min)		
Recovery indicator	Group I	Group II	Group III
First movement	12.5 ± 8.2	12.6 ± 10.5	10.5 ± 3.5*
Extubation	11.6 ± 5.8	11.2 ± 4.1	7.5 ± 1.0
Sternal recumbency	34.7 ± 23.8	37.4 ± 14.1	27.2 ± 2.3
Standing	292.2 ± 14.7	71.6 ± 20.1*	79.3 ± 15.9*

*Significantly (*P* < .05) different from values obtained for pigs undergoing laparoscopy.
